# Use of telehealth for measurement of anthropometrics in toddlers and their parents

**DOI:** 10.3389/fdgth.2025.1548607

**Published:** 2025-07-04

**Authors:** Sarah S. Farabi, Cindy Schwarz, Bria Lee-Robinson, Lauren G. Fiechtner, Victor Davila-Roman, Rachel G. Tabak, Debra Haire-Joshu

**Affiliations:** ^1^ Goldfarb School of Nursing, Office of Nursing Research, Saint Louis, MO, United States; ^2^ Department of Medicine, Division of Nutritional Science & Obesity Medicine, Washington University School of Medicine in St. Louis, Saint Louis, MO, United States; ^3^ WashU Public Health, Washington University in St. Louis, Saint Louis, MO, United States; ^4^ Division of Gastroenterology and General Academic Pediatrics, Mass General for Children, Boston, MA, United States; ^5^ Department of Medicine, Division of Cardiology, Washington University School of Medicine in St. Louis, Saint Louis, MO, United States

**Keywords:** anthropometrics, reliability, toddler, parent, virtualtelehealth

## Abstract

**Introduction:**

The use of telehealth (e.g., live video calling) to collect anthropometric data in toddlers and their parents to increase participation in lifestyle interventions holds promise. But, there is limited evidence to support reliability of telehealth for the collection of these measures. This study aimed to determine the reliability of use of telehealth with parents to collect anthropometric and blood pressure measures on themselves and anthropometric measures on their toddler and determine the acceptability of instruction.

**Methods:**

This cross-sectional study was conducted as part of the developmental phase of a larger study. Research staff instructed parents via video call to measure their own and their toddler's anthropometrics, and their own blood pressure. Next, research staff collected the same measurements in-person. Intraclass Correlation Coefficients (ICC), relative technical error of measurement (TEM) and reliability coefficient values were computed.

**Results:**

Thirty-seven parent/toddler dyads were enrolled in the study. ICC values for parent vs. research staff measured parental height and weight were 0.98 and 0.99, respectively, and relative TEM values were 0.44% and 0.14%, respectively. ICC values for parent vs. research staff measured toddler height and weight were 0.98 and 0.99, and relative TEM values were 1.60% and 0.82%, respectively. ICC values for parent vs. research measured systolic and diastolic blood pressure were 0.86 and 0.89 respectively.

**Discussion:**

Parental self-measurement of height, weight, and blood pressure, and measurement of toddler height and weight is reliable and acceptable to parents when performed using telehealth.

## 1 Introduction

Cardiometabolic disease, such as obesity and hypertension, are increasing in people of childbearing age at alarming proportions. This is particularly true among people from underrepresented minority (URM) populations in research as defined by the National Institutes of Health (i.e., low socioeconomic status, racial/ethnic minorities, rural communities) (1–4). It is estimated that at least 25% of people who become pregnant have obesity, with higher rates seen in racial and ethnic minorities (2). Hypertensive disorders of pregnancy affect ∼15% of pregnancies with higher rates seen in URM populations (5, 6). This is problematic as hypertensive disorders of pregnancy are associated with complications including preterm birth and are the leading cause of pregnancy-related death in the United States. Cardiometabolic disease in pregnancy also confers an increased risk for disease in the offspring (7–9).

There is a critical need to develop and test effective strategies to mitigate cardiometabolic disease, particularly in URM populations. However, barriers to in-person data collection limit opportunities for participation in research that tests lifestyle interventions. This is particularly true for parents and their toddlers due to barriers such as time and transportation (10). URM tend to experience more barriers to attending required in-person visits in lifestyle intervention studies due to competing demands, time constraints, and transportation barriers, leading to studies without generalizability to URM populations (11, 12).

With the recent widespread use of telehealth (e.g., live video calls) there is an opportunity to conduct research assessments remotely, potentially mitigating time and transportation constraints to participation (10, 13). However, there have been limited studies to investigate the reliability of instruction using telehealth for parental self-measurement of blood pressure, height, and weight, and parental measurement of toddler height and weight (14–17).

The Early Intervention to Promote Cardiovascular Health of Mothers and Children (ENRICH) Collaborative is a multisite trial funded by the National Heart, Lung, and Blood Institute (NHLBI; grant UG3 HL162970) (18). The study described in this paper was conducted as part of the 2-year developmental phase of ENRICH to inform measure selection and intervention development for use in the larger implementation phase of the trial.

The purpose of this study was to (1) determine the reliability of instruction via telehealth for self-measurement of parental height, weight, and blood pressure compared with measurement by trained research staff; (2) determine the reliability of instruction via telehealth of parental measurement of toddler height and weight compared with measurement by trained research staff, and; (3) determine the acceptability of instruction via telehealth of the parent and toddler height and weight measures.

## 2 Methods

### 2.1 Study design

**Figure 1:**
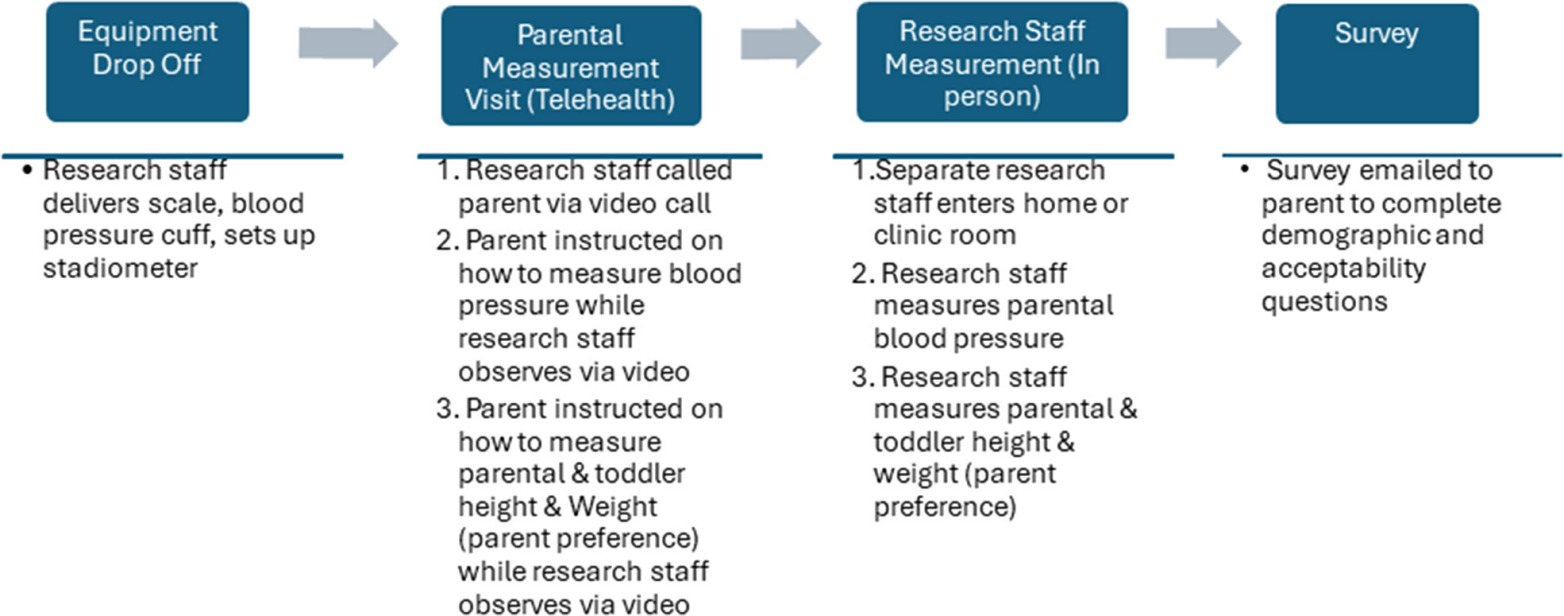
Overview of the study process.

A cross-sectional study design was used as both visits, the remote (parental measurement visit) and in-person (research staff measurement visit) were conducted on the same day. The parental measurement visit via telehealth always occurred first to avoid training effects of the participants. Figure 1 provides an overview of the study process.

### 2.2 Recruitment of sample and overview of study visits

Parents were recruited from a local obstetrics clinic and through a local parents' group on social media. All study procedures were approved by the Washington University in St. Louis Institutional Review Board prior to starting the study. Oral and written informed consent was obtained from all participants prior to starting study procedures. To be eligible to participate, the parent had to have a child aged 2 years or younger (eligible up until week before 3rd birthday). Only a single parent was required to participate. There were no additional inclusion/exclusion criteria. Informed consent was obtained before the start of data collection. The parent was then asked to complete a brief survey online about their demographics (i.e., race, ethnicity) and acceptability of the process at the end of the visit. Table 1 defines the two visits and Figure 1 provides an overview of the study process. Anthropometric protocols for the parent and toddler were developed based on guidelines from the World Health Organization and Centers for Disease Control (19–21), the protocol for parental blood pressure was developed in accordance with the American Heart Association and International Society of Hypertension Global Hypertension Practice Guidelines (22, 23). The parental measurement visit occurred first and the research measurement visit started within 10 min of the completion of the parental measurement.

**Table 1:** Definition of study visits.

Name of visit	Definition
**Parental measurement (telehealth) visit**	Research staff member instructed parent on measurements for themselves and their toddler while observing the measurements via telehealth (video call).
**Research staff measurement visit**	Research staff member collected parent and toddler measurements in person either in home or clinic setting.

### 2.3 Parental measurement (telehealth) visit

Prior to the start of the visit, a research staff member entered the home (*n* = 27) or clinic room (*n* = 10) and provided the parent with a stadiometer, a weight scale and automated blood pressure device (monitor and cuff). Next, a research staff member (from another room in the clinic or outside of the home) called the participant via video call on their phone or a laptop device (provided by the study team). Via video call, the research staff member instructed the parent to complete the measures (height, weight, blood pressure) and how to position the phone or laptop so they could observe the parent completing the measures. Blood pressure was collected first, then either toddler height and weight or parental height and weight were measured depending on parental preference.

### 2.4 Research staff measurement visit

Immediately after the parental measurement visit was complete, a second research staff member blinded to the readings from the parental measurement collected blood pressure, height, and weight following the same protocols (19–23) in either in the clinic or in the home. Similar to the parent measurement visit, blood pressure was measured first and then either toddler height and weight or parental height and weight were measured depending on parental preference.

### 2.5 Study measures

#### 2.5.1 Parental blood pressure

An automated blood pressure device (OMRON Model 3™) was used to collect parental blood pressure in their left arm. Before measuring the parent's blood pressure, the parent was asked to sit for 5 min and keep as quiet as possible prior to the measurement of the blood pressure. Blood pressure was measured twice, if the systolic or diastolic pressure readings were different by more than 10 mmHg, a third measurement was taken. The average of the two (or three) readings was taken.

#### 2.5.2 Parental height

Parental height was measured with a portable stadiometer (Seca 213). The parent was asked to remove their shoes and any hair ornaments from the top of their head. The parent was asked to stand up straight against the stand with their body weight evenly distributed on both feet, look straight ahead, shoulders relaxed, arms at their sides, legs straight and knees together with feet flat on the platform heels together, toes slightly apart. After the position was confirmed, either the parent (parental measurement visit) or research staff (research staff measurement visit) moved the head plate, so it rested firmly on the top of the head. The parent was asked to stand up tall, take a deep breath and hold the position, and then, bend their knees and step away from stadiometer. The reading was recorded by research staff. Parent height was recorded in feet, inches, and fraction (to the 1/8th inch) and converted to meters using a standardized conversion formula (length [in] × 0.0254).

#### 2.5.3 Parental weight

The parent placed the scale (Patient Aid PA-550XL Portable Medical Scale) on a hard flat surface (non-carpeted) and the parent was asked to remove shoes and any heavy clothing such as a coat or sweater. The scale was turned on and zeroed. Once zeroed, the parent was asked to stand on the scale and wait for the recording to stabilize. Weight was recorded in pounds to the first decimal place and converted to kilograms (kg) to the first decimal place using a standardized conversion formula (weight [pounds]/2.2).

#### 2.5.4 Toddler length/height

For toddlers under two and who were not able to stand independently, toddler length was taken using an infant length board (Seca 210™). A second adult was asked to assist the parent during the telehealth visit. The length board was placed on a sturdy, flat surface (i.e., medical table or floor). The toddler's body was positioned so that the head was touching the flat plastic plate at the top of the length board. The knees were pressed firmly down on the mat and length was obtained with the feet at 90 degrees. For toddlers two years old and able to stand, height was measured with the stadiometer. The toddler was asked to put their feet up against the wall and stand up straight against the backboard with her body weight evenly distributed on both feet, looking straight ahead, shoulders relaxed, arms at her sides, legs straight and knees together with feet flat on the platform heels together, toes slightly apart. The research staff or parent helped to hold the head straight and keep eyes looking forward. After position was confirmed, either the parent (parental measurement visit) or research staff (research staff measurement visit) moved the head plate so it rested firmly on the top of the toddler's head. The toddler was asked to stand up tall, take a deep breath and hold the position, and then, to bend their knees and step away from stadiometer. The length or height was measured twice and the average was taken and was recorded in centimeters to the first decimal place.

#### 2.5.5 Acceptability survey

Parents were asked to complete a brief survey about the data collection process. Questions included two open ended questions “What did you like about the process?”, and “How could the instructions be improved?”, and one yes/no question, “Would you recommend others to do this study?”.

### 2.6 Statistical analysis

Average values for parental height, weight, and blood pressure and toddler height met assumptions for normality and were computed for the parental measurement and research staff measurement visits and compared using paired *t*-tests. Toddler weight was compared using a Wilcoxon signed-rank test since the data were not normally distributed. Two-way random-effects model Intraclass Correlation Coefficients (ICC) values were computed to determine the reliability between the parental and research staff measurements. ICCs range from 0 to 1, with values indicating <0.5: poor; 0.5–0.75: moderate; 0.75–0.9: good; and >0.9: excellent agreement (24). Bland-Altman plots were also used to assess the reliability of the measures (25, 26). Further, to calculate the accuracy of height and weight measurements for both parent and toddler, the technical error of measurement (TEM) and Reliability coefficients were calculated (27). TEM is an accuracy index (27–29), lower TEMs indicate higher accuracy. A relative TEM of <2.0% indicates acceptable accuracy. The Reliability coefficient indicates consistency in agreement between two examiners, values range from 0 to 1, with values closer to 1 indicating higher reliability (27–29). Statistical analyses were performed by using STATA/IC 16.0 for Windows (STATA Corp LLC, Texas, USA).

## 3 Results

A total of 37 parent/toddler dyads enrolled in the study. Table 2 provides demographics of parents and toddlers. Of the 37 dyad visits, one (2.7%) parent/toddler pair was excluded due to difficulties with the telehealth measures (i.e., video was too blurry to see measurements) for height and weight. Four (10.8%) blood pressure readings were excluded, two were excluded due to not having a large enough cuff to fit properly, and an additional two were excluded because the toddler was crying loudly during the research staff measurement visit and not the parental measurement visit. One (2.7%) parental height and weight measurement was missing for the parental visit as the data were not saved on the server. Four (10.8%) toddler height measurements were excluded: two (5.4%) were excluded due to poor positioning of the camera which prevented the research team from adequately instructing and visualizing the parent during measurement of height/length, and two (5.4%) height recordings were excluded as the toddler would not stand or lay still for the measurement for either or both of the visits (1 for both the parental and research staff visits and the other for only research staff visit). Supplementary Table 1 provides an overview of key technical challenges with potential solutions and Table 3 provides the number included for each measurement.

**Table 2:** Demographics of sample.

Sample demographics	*n* = 37
Parent age, years, mean (SD)	30.8 (5.8)
Toddler age, months, mean (SD)	21.7 (6.8)
Parent race, *n* (% total)	
Black or African American	15 (40.5)
White	20 (54.1)
Asian	1 (2.7)
Prefer not to say	1 (2.7)
Parent Ethnicity, *n* (% total)	
Not Hispanic or Latino	33 (89.2%)
Not reported	4 (10.8%)
Parent education, *n* (% total)	
Some or less than High school	2 (5.4)
High school diploma or GED	9 (24.3)
Some college or Technical school	4 (10.8)
College degree or higher	19 (51.4)
Not reported	3 (8.1)
Parent hypertension diagnosis, *n* (% total)	5 (13.5)
Parent history of gestational diabetes, *n* (% total)	3 (8.1)

**Table 3:** Average values and reliability metrics for parent and toddler height and weight and parent blood pressure.

Measure	Research staff measurement	Parental measurement (Telehealth)	Average difference^a^	Relative TEM (%)	R-coefficient	ICC
Parent						
Height, m (*n* = 36)	1.63 (0.06)	1.64 (0.06)	0.007 (0.008)	0.44	0.98	0.98 (0.96–0.99)
Weight, kg (*n* = 36)	86.5 (23.1)	86.5 (23.1)	0.11 (0.13)	0.14	0.99	0.99 (0.99–0.99)
Systolic BP, mmHG (*n* = 33)	109.7 (9.2)	111.1 (10.9)	5.4 (4.6)	-	-	0.86 (0.72–0.93)
Diastolic BP, mmHG (*n* = 33)	74.6 (8.8)	74.2 (7.9)	3.4 (4.0)			0.89 (0.78–0.95)
Toddler						
Height, m (*n* = 32)	0.837 (0.067)	0.841 (0.066)	0.0114 (0.0154)	1.60	0.96	0.98 (0.96–0.99)
Weight, kg (*n* = 36)	12.4 (2.5)	12.4 (2.5)	0.07 (0.13)	0.82	0.98	0.99 (0.99–0.99)

^a^ Average of absolute value of differences; data are mean (SD) or value (95% CI).

### 3.1 Parental results

**Figure 2:**
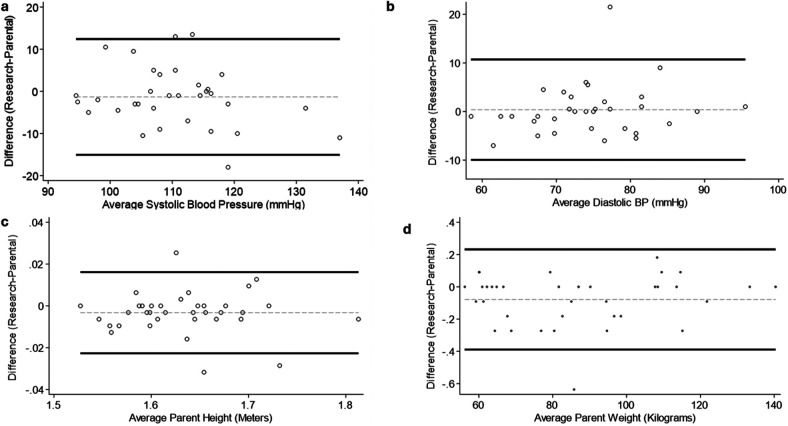
Bland–Altman plots for parent measures. **(a)** Systolic Blood Pressure (mmHg); **(b)** Diastolic Blood Pressure (mmHg); **(c)** Height (m); **(d)** Weight (Kg). Circles indicate individual measures, black lines indicate 95% Confidence Intervals, gray dashed line indicates average difference.

The Bland-Altman Plots for parental systolic and diastolic blood pressure, height, and weight are shown in Figure 2. The plots show good agreement between systolic and diastolic blood pressure readings, and very good agreement for height and weight for the parental vs. research staff measurements. ICC values for systolic and diastolic blood pressure were 0.86 and 0.89 respectively, and ICC values for parental height and weight were 0.98 and 0.99 respectively, indicating excellent reliability for between parental and research staff measurements (Table 3). Relative TEM values for parental height and weight were 0.44 and 0.14%, respectively, and reliability coefficients 0.98 and 0.99, respectively, demonstrating acceptable accuracy between parental and research staff measurements (Table 3). The average values for parental and research staff measurement visit were not significantly different for height (*p* = 0.22), weight (*p* = 0.32), or systolic or diastolic blood pressure (*p* = 0.24 and *p* = 0.41, respectively). The average value of differences between parental systolic blood pressure was 5.4 ± 4.6 mmHg, diastolic blood pressure was 3.4 ± 4.0 mmHg, weight was 0.11 ± 0.13 kg, and parental height was 0.007 ± 0.008 m (Table 3).

### 3.2 Toddler results

**Figure 3:**
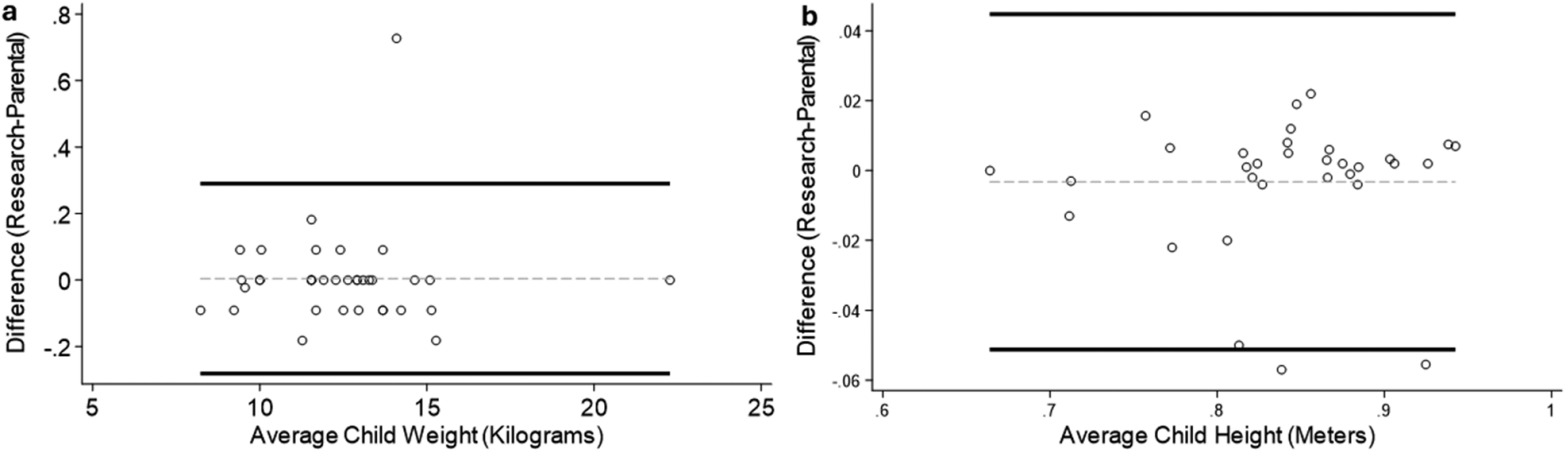
Bland–Altman plots for toddler measures. **(a)** Height (m); **(b)** Weight (Kg). Circles indicate individual measures, black lines indicate 95% Confidence Intervals, gray dashed line indicates average difference.

Figure 3 shows the Bland-Altman Plots for toddler height and weight. The plots show very good agreement between parental and research staff measurements. ICC values for Toddler height and weight were 0.98 and 0.99, respectively, indicating excellent reliability between parental and research staff measurements (Table 3). Relative TEM values for toddler height and weight were 1.60 and 0.82%, respectively, and Reliability coefficients 0.96 and 0.99, respectively, showing acceptable accuracy between parental and research staff measurements (Table 3). The average values for the parental and research staff measurements were not significantly different for height (*p* = 0.25) or weight (*p* = 0.37). The average absolute difference between the parental and research visits for toddler length/height measurements was 0.011 ± 0.015 meters and weight was 0.07 ± 0.13 kg (Table 3).

### 3.3 Acceptability

Participants found the process acceptable. Of the 33 people who completed the acceptability survey, all 33 said that they “would recommend the process to others”. When asked for how to improve the instructions or the process, 3 people suggested that pictures or written instructions would help in addition to accompany the verbal instructions, and the remaining recommended no changes. The majority of the participants found the process easy; 7 people mentioned that they liked not having to leave their house, and 16 people reported that it was “easy” or “simple” to do.

## 4 Discussion

The use of telehealth to collect anthropometric data in toddlers and their parents may increase participation in lifestyle interventions, but the validation of telehealth for the collection of these measures remains limited. The present study found very good reliability for instruction via telehealth (live video call) of parental measurement of height and weight for toddlers and self-measurement of the parents relative to measurement by trained-research staff. We found good reliability between parental and research staff measurements for blood pressure. Lastly, we found that instruction via telehealth was acceptable to parents of toddlers. Our findings are in-line with previous studies in older children and adults demonstrating reliability of parental self-measurement of height and weight and parental instruction for measurement of child height and weight (14–17, 30). However, to our knowledge, this is the first study establishing the reliability of instruction via telehealth for measurement of height and weight among toddlers.

Previous studies have focused on older children demonstrating reliability of parental measurement of height and weight (14, 15, 17), but studies are needed to assess accuracy of measurement of toddlers (ages 1–3). Toddlers can be more difficult to measure in the clinic due to inability to follow directions and/or being unwilling to lay or stand still for measurement. Measuring children in their own home by their parents with whom they are comfortable may result in more accurate results. Similar to previous studies, very good agreement was found for toddler height and weight measurement between research staff and parents. Button et al. found no significant differences between height and weight measured in-person with trained research staff and by the parent via telehealth (live video calling) in children ages 6–15 years old. We found higher reliability for our weight measurements compared to those reported by previous investigations. However, our study visits occurred within 10 min of each other while there was up to a week between measurements in the previous studies. Similar to previous studies (14, 16, 17), we used remote observation in our study. Zhang et al., and Button et al., used a less expensive measurement method (tape measure) for height which resulted in non-significant differences for height in slightly older children (14, 17). Our results indicate that with standard measurement tools and telehealth, parents can reliably measure their toddler's height and weight remotely. This expands opportunities for participation in lifestyle interventions for those facing barriers to attending in-person visits at research centers.

Remote measurement of blood pressure is common in clinical practice as well as research studies (30–34). Our findings demonstrated good agreement between self-measurement of blood pressure and blood pressure measured by research staff in parents. Of note, the reported accuracy of the Omron blood pressure monitor is ±3 mmHg or 2 percent per the manufacturers website (35). Another important finding in our study is that parents reported a high degree of acceptability with the instruction via telehealth and noted that they were particularly happy that they did not have to leave their home to participate in the study. This is important as a commonly cited barrier to participating in pediatric research is the logistical burden of participation, particularly in URM families (10, 36). This finding also extends previous reports that remote monitoring of blood pressure clinically is acceptable and feasible (37). Our results indicate cardiometabolic outcomes in parents and toddlers can reliably be measured remotely in research trials aimed at decreasing cardiometabolic disease. Thus, findings from this study can be used to justify use of telehealth approaches to data collection which may help to alleviate logistical burden of research study participation for URM populations.

Major limitations of our study include that the study is cross-sectional so temporal changes cannot be assessed, the sample size is small, and height and weight were measured using the imperial scale and then converted to the metric scale which could influence results. This was a single site and individuals who are more comfortable with technology or had better internet connection may have self-selected to be in the study limiting the generalizability of the findings to broader populations. Further, the equipment including a stadiometer, scale, and blood pressure cuff was provided to parents at their home. For fully remote measurements this equipment would need to be mailed and addition of more simple equipment such as tape measures or home blood pressure cuffs would help to decrease the challenge of equipment provision. However, as previously stated, other studies have shown that parents can reliably measure height using tape measurements in older children (14, 17). Future studies should investigate the reliability of measuring height using tape measurements in toddlers. Other limitations include that a dynamic error propagation model using statistical or machine learning techniques to predict and compensate for potential inaccuracies in real time was not applied and no algorithmic frameworks for scaling to larger populations or predicting measurement success based on contextual variables (e.g., lighting, camera resolution, child cooperation level) were used.

In summary, we found good reliability between parental and research staff measurement of height, weight, and blood pressure in parents and height and weight in their toddlers. Our findings support that use of instruction via telehealth is feasible and acceptable for parents of toddlers and may decrease barriers for participation in lifestyle intervention research studies aimed at decreasing cardiometabolic diseases in parents and their young children.

## Data Availability

The raw data supporting the conclusions of this article can be obtained from the corresponding author upon request.
