# Supplementary material for: Use of telehealth for measurement of anthropometrics in toddlers and their parents
Source: Front Digit Health. 2025 Jul 4;7:1548607. doi: 10.3389/fdgth.2025.1548607 (PMC12271149; doi:10.3389/fdgth.2025.1548607)
Supplement: Supplementary file 1 [file Table1.docx]

**Supplemental Table 1. Technical Challenges in Study and Potential Solutions**

| **Technical Challenge** | **Possible Solution** |
| --- | --- |
| **Unable to visualize measures** | - Prior to starting measurements, have parent move camera to new location so staff can visualize where measures occurring. - For infant length, place camera at top of length board where staff can visualize both head and feet placement. |
| **Disrupted connection** | - Ask participant to take pictures of the measures if video not clear. |
| **Blood pressure cuff not big enough** | - Have parent measure arm with tape measure prior to sending cuff. |
| **Crying toddler** | - Reschedule visit if possible - Change order of measurements to take BP when toddler is calm - Schedule visit at time of day that is “best” for child (i.e. earlier in morning or after nap) |
| **Data not saved on server** | - Record measurements on paper and computer if possible |
